# Comparative pathogenomics of *Clostridium tetani*

**DOI:** 10.1371/journal.pone.0182909

**Published:** 2017-08-11

**Authors:** Jonathan E. Cohen, Rong Wang, Rong-Fong Shen, Wells W. Wu, James E. Keller

**Affiliations:** 1 Laboratory of Respiratory and Special Pathogens, Center for Biologics Evaluation and Research, Food and Drug Administration, Silver Spring, Maryland, United States of America; 2 Facility for Biotechnology Resources, Center for Biologics Evaluation and Research, Food and Drug Administration, Silver Spring, Maryland, United States of America; Aarhus Universitet, DENMARK

## Abstract

*Clostridium tetani* and *Clostridium botulinum* produce two of the most potent neurotoxins known, tetanus neurotoxin and botulinum neurotoxin, respectively. Extensive biochemical and genetic investigation has been devoted to identifying and characterizing various *C*. *botulinum* strains. Less effort has been focused on studying *C*. *tetani* likely because recently sequenced strains of *C*. *tetani* show much less genetic diversity than *C*. *botulinum* strains and because widespread vaccination efforts have reduced the public health threat from tetanus. Our aim was to acquire genomic data on the U.S. vaccine strain of *C*. *tetani* to better understand its genetic relationship to previously published genomic data from European vaccine strains. We performed high throughput genomic sequence analysis on two wild-type and two vaccine *C*. *tetani* strains. Comparative genomic analysis was performed using these and previously published genomic data for seven other *C*. *tetani* strains. Our analysis focused on single nucleotide polymorphisms (SNP) and four distinct constituents of the mobile genome (mobilome): a hypervariable flagellar glycosylation island region, five conserved bacteriophage insertion regions, variations in three CRISPR (clustered regularly interspaced short palindromic repeats)-Cas (CRISPR-associated) systems, and a single plasmid. Intact type IA and IB CRISPR/Cas systems were within 10 of 11 strains. A type IIIA CRISPR/Cas system was present in two strains. Phage infection histories derived from CRISPR-Cas sequences indicate *C*. *tetani* encounters phages common among commensal gut bacteria and soil-borne organisms consistent with *C*. *tetani* distribution in nature. All vaccine strains form a clade distinct from currently sequenced wild type strains when considering variations in these mobile elements. SNP, flagellar glycosylation island, prophage content and CRISPR/Cas phylogenic histories provide tentative evidence suggesting vaccine and wild type strains share a common ancestor.

## Introduction

*Clostridia* are a heterogeneous genera of saprophytic, gram-positive, spore-forming anaerobes comprised of at least 209 species and 5 subspecies [1]. Although primarily found in soil, *Clostridia* are commonly found among gut flora. Pathogenic species such as *Clostridium botulinum*, *C*. *perfringens*, *C*. *difficile* and *C*. *tetani* each produce one or more unique exotoxins that sicken the host and can eventually result in death [2]. Most other *Clostridia* are non-pathogenic.

Diversity within a species can be significant. For example, although tetanus neurotoxin (TeNT) is closely related to botulinum neurotoxins, more than 100 *C*. *botulinum* strains have been sequenced, annotated, and organized into 4 distinct clusters expressing 7 distinct neurotoxins and 2 “chimeric” toxin hybrids [3, 4]. For *C*. *tetani* phylogenomic analysis of protein, 16s ribosomal RNA, TeNT gene (TetX) and TetX transcriptional regulator (TetR) gene homology show *C*. *tetani* is most closely related to Group I proteolytic *C*. *botulinum* strains [5]. Unlike TeNT, botulinum neurotoxins form large protease-resistant complexes comprised of neurotoxin bound to bacterial hemagglutinin and non-hemagglutinin proteins, which protect the toxin in the digestive tract and aid its transport from gut to blood [6, 7]; toxin-related gene clusters in the botulinum family are moderately conserved with about 40% identity but have diverse locations in the genome, either on chromosome, plasmid, or bacteriophage loci depending on the *C*. *botulinum* species [2, 4]. *C*. *tetani* is simpler, carrying a single plasmid encoded with TetX and TetR but without genes for toxin-bound accessory rendering TeNT non-toxic via ingestion [8].

Tetanus toxoid (TT), a non-toxic form of TeNT is used for the prevention of tetanus disease. The *C*. *tetani* vaccine strain is derived from a laboratory strain collected by G.W. McCoy around 1917–1919 at the US Public Health Service, Hygienic Laboratory [9–12]. The strain is commonly referred to as the “Harvard strain” and was distributed among researchers and manufacturers beginning in the early 1920s and continueing throughout the 1950s [13]. It continues to be used around the world for TT manufacture. Our genomic analysis demonstrates the vaccine strain is a clade derived from the same progenitor. Three different Harvard strains used mainly by European manufacturers and laboratories (strains E88, CN655 and “Strain A”) have been sequenced [14, 15].

Our study aimed to determine the extent of genetic drift that may exist between US and European Harvard strains following decades of separate handling and passaging. We also sought to compare genetic diversity between vaccine strains and several wild-type *C*. *tetani* strains. High-throughput DNA sequencing was performed using our in-house Harvard strain “C2” which was originally obtained from a US vaccine manufacturer in 1962 and has been stored lyophilized since 1965. We also sequenced strain ATCC 19406, which is a diagnostic laboratory strain and two wild-type *C*. *tetani* strains, ATCC 453 and ATCC 9441. Previously published genomic data from seven other *C*. *tetani* strains, [14–16] were used for comparative analyses (Table 1). We discuss similarities and differences among these 11 strains in terms of single nucleotide polymorphisms (SNP) and mobile elements. The analyses indicate that Harvard vaccine strains are remarkably similar separated by fewer than 300 SNPs acquired across all the genomes. No mutations were observed in TetR or TetX providing assurance that genetic drift has not compromised tetanus toxin antigenicity used in vaccine manufacture.

**Table 1:** Genetic analysis of sequenced strains.

Strain	Genome (Mb)	#ORF	N50 (#Contig)	Plasmid (bp)	#ORF	Notes	Geographic point of origin
**E88 (UK)**	2.80	2640	-	74,082	93	Harvard	North America
**C2 (USA**	2.76	2840	263,508 (32)	72,569	94	Harvard	North America
**Strain A**	2.75	2862	96,331 (90)	71,728	90	Harvard	North America
**CN655**	2.78	2919	110,001 (111)	69,697	88	Harvard	North America
**ATCC 19406**	2.79	2799	181,872 (48)	-	-	Harvard	North America
**ATCC 9441**	2.72	2786	1,734,923 (29)	80,540	95	WT	North America
**ATCC 453**	2.80	2874	253,841 (37)	90,995	115	WT, feces	China
**ATCC 454**	2.84	2867	87,223 (92)	62,231	85	WT, feces	China
**GTC-14772**	2.88	3053	135,714 (125)	64,979	78	WT, wound	Japan
**12124569**	2.81	2717	-	58,369	77	WT, wound	France
**184.08**	2.84	3033	70,366 (135)	70,217	88	WT, wound	France

## Materials and methods

### Bacterial strains, media, and growth conditions

*Clostridium tetani* strains were obtained through the American type culture collection (ATCC) and maintained as glycerol stocks at -70°C. Strain C2, obtained by CBER in 1962 from a commercial vaccine manufacturer, was passaged once in 1965 and stored lyophilized in heat-sealed glass vials. Bacterial culture stocks were propagated in thioglycollate media containing resazurine (Sigma Aldrich, USA) in anaerobic boxes (BioMerieux, Craponne, France) at 35°C. For tetanus toxin expression, 24 hour cultures were inoculated into Massachusetts medium as originally described in Latham et al. [17] containing NZ-Case TT (Kerry Bio-Science, Beloit WI) and harvested after 6 days. For swarming behavior and motility, 24 hr cultures were plated onto 1.5% Trypticase soy agar plates (TSAII, BD Biosciences, Franklin Lakes, NJ) containing 5% defibrillated sheep blood (Hemostat Laboratories, Dixon CA) and incubated at 30°C for 48 hrs before colonies were imaged.

### Bacteriophage induction, isolation and purification

Ten ml *C*. *tetani* cultures were transferred to sterile 10 cm glass petri dishes and UV irradiated for 60 sec with gently swirling every 10 sec. UV- and mock-treated samples were diluted 1:1 with TG media, and incubated at 35°C for 24 hrs under anaerobic conditions. A 600 μl sample was removed and bacterial density was measured at OD_600_ on a Beckman DU-530 spectrophotometer (Beckman Coulter, Inc. Brea, CA) to monitor phage induction and lysis. The following day, culture supernatants were collectedfollowing two stages of centrifugation, first at 3,500 x g for 20 min then twice at 64,000 x g for 90 min to collect phage particles. Supernatants were discarded, pellets were resuspended in either sterile H_2_O (for transmission EM) or phage buffer (TM10, 10 mm Tris-Cl, pH 7.5 10 mM MgCl_2_, 1 mM CaCl_2_).

### Bacterial genomic and phage DNA isolation

Frozen glycerol stocks of *C*. *tetani* strains were inoculated into TG media and propagated for 24 hrs. Genomic DNA was isolated using a DNeasy Blood and Tissue kit (Qiagen, Germantown MD) according to the manufactures instructions with slight modifications. For genomic DNA isolation, 5 ml of a 24 hr culture was centrifuged at 3,500 x g for 20 min and cell pellets were rinsed in sterile-filtered STE buffer (100 mM NaCl, 10 mM Tris-Cl, pH 7.5, and 1 mM EDTA), re-suspended in 1.5 ml STE buffer and re-centrifuged for 5 min at 12,000 x g. Pellets were then resuspended in 200 μl STE containing 10 mg/ml lysozyme (Sigma, St. Louis MO) and incubated at 37°C for 60 min, followed by 5 min RNase treatment at room temperature. RNase-treated samples were then processed for genomic DNA. Genomic DNA quality was measured on a Qubit fluorometer (Invitrogen, Carlsbad CA). Phage DNA was purified from concentrated phage particles using a Phage DNA isolation kit according to manufactures instructions (Norgen Biotek, Ontario, Canada). Phage PCR was performed on an Applied Biosystems Veriti Thermal Cycler (Invitrogen, Carlsbad CA) using PCR SuperMix (Invitrogen, Carlsbad, CA) according to manufactures recommendations with phage-specific primers (S5 Table).

### Next-generation sequencing and analysis

Genomic and phage DNA sample libraries were prepared using either a Nextera or Nextera XT sample preparation kit according to manufactures’ instructions and sequenced on a MiSeq in triplicate kit (Illumina, San Diego CA). Genome assembly against *C*. *tetani* E88 genome [14] was performed using ABySS genome assembler [18] and de novo assembly of paired read data was performed using CLC Genomics Workbench (CLC Bio, Aarhus Denmark). Sequence data was generated from 3 separate sequencing runs using 3 different DNA preparations from each strain. De novo assembly of paired read data and direct assembly against the E88 scaffold were performed as ways to improve confidence in sequence accuracy and for increased reliably when comparing multiple genomes [19]. Contigs were then aligned and reorganized against *C*. *tetani* E88 reference strain with MAUVE [20]. Single nucleotide polymorphisms (SNPs) were called in MAUVE against aligned contigs from sequenced strains; multiple SNP comparisons between strains was represented by four-way Venn diagrams using the program Venny [21] Pairwise BLAST was performed using standalone BLAST [22, 23] and displayed using BLAST Ring Image Generator, BRIG [24]. Genomic relationship of sequenced *C*. *tetani* strains against E88 reference strain were calculated by BLAST average nucleotide identity method, ANIb in Jspecies [25].

Prophage insertions within genomic regions were identified using the phage search tool, PHAST http://phast.wishartlab.com/index.html [26]. GC skew was calculated for each predicted prophage regions using the java applet GSkew (http://genskew.csb.univie.ac.at/). 16sRR sequences were retrieved for phylogenetic analysis of bacterial strains that matched to predicted phage, CRIPSR/Cas, or predicted proteins within the flagellar glycosylation island (S3 Fig and S1 Table). For each phage protein (integrase, portal protein, tail-tape measure protein, and TerL large terminase), pairwise BLAST was performed to identify top 10 scoring matches when possible. Protein sequences with % identity scores less than 50% were discarded. Non-redundant protein sequences were then aligned in MEGA version 6 [27] using the MUSCLE algorithm. Phylogenetic trees and bootstrapping (500 iterations) was performed using maximum likelihood analysis and displayed as circular phylogenetic trees. For each set of conserved phage proteins, phylogenetic relationships between orthologous genes were inferred by BLAST, multiple sequence alignment, and maximum likelihood analysis. Bacterial species were then binned according to either environmental isolate (soil and sediment, fecal-oral, waste and run-off water, and fermentation) or species in the case of *C*. *botulinum*, *C*. *difficile*, *C*. *perfringens* and *C*. *tetani* strains.

All genome data used in this study are available through GenBank/NCBI. Accession numbers for *C*. *tetani* strains and phages sequenced in this study follow.

Strain C2: BioProject PRJNA260192, accession # JRGG01 and assembly #GCA_000762335.1. Strain C2 phage assembly: Accession KM983333.1 and KM983334.1). Strain ATCC 9441: BioProject PRJNA260193, accession # JRGH01 and assembly GCA #_000762315.1. ATCC 9441 phage assembly KM983329.1.

Strain ATCC 19406: BioProject PRJNA260197, accession # JRGJ01 and assembly # GCA_000762305.1. ATCC 19406 phage assembly KM983330.1, KM983331.1, KM983332.1. Strain ATCC 453: BioProject PRJNA260198, JRGI01, assembly # GCA_000762325.1 and WGS # LBNB01. Strain ATCC 453 phage assemblyKM983327.1, KM983328.1. Sequence data from additional strains were obtained for the following strains. Strain E88: BioProject PRJNA81 and assembly # GCA_000007625.1, accession #AE015927.1 and AF528097.1. Strain ATCC 454: BioProject PRJNA158341, LBNB01 and assembly # GCA_000987095.1. NIH Japan Strain GTC 14772 (SRA DRX014318). Strain 12124569 PRJEB4542 and assembly # GCA_000967115.1, HG530135.1 and HG530136.1. Strain A: BioProject PRJNA265068 and assembly # GCA_000818755.1 and WGS # JWIX01. Strain 184.08: BioProject PRJNA265068 and assembly # GCA_000805775.1 and WGS # JSWD01. Strain CN655: BioProject PRJNA265068 and assembly GCA_000805755.1 and WGS # JSWC01.

CRISPR arrays in C. tetani genomes were identified by CRISPRFinder (http://crispr.u-psud.fr/Server/) [28]. CRISPR spacer targets were identified by CRISPRTarget (http://bioanalysis.otago.ac.nz/CRISPRTarget/) [29]. CRISPR spacer arrays for 11 strains were aligned with each other and conservation heatmaps were calculated based on rank order of sequence identity across each spacer array. CRISPR spacers for each strain were ranked by sequence identity to phage- and/or plasmid derived sequences across bacterial species and sorted based on overall occurrence (for all strains). CRISPR spacers identified in *C*. *tetani* strains corresponded primarily to phage sequences in other *C*. *tetani* strains.

### EM analysis of *C*. *tetani* phage particles

50 μl of 0.5% (g/vol) bacitracin (Sigma, St Louis MO) was added to purified phage samples prior to staining to act as a wetting agent [30]. Formvar-coated grids (EM Services, Hatfield, PA) were treated with 0.1% poly-lysine to increase phage binding. 10 μl of phage solution and was added and allowed to dry for 5 min, followed by negative staining with 2% (g/vol) sterile-filtered uranyl acetate (Sigma, St Louis MO) for an additional 60 sec. Grids were rinsed in distilled water and dried for 5 min prior to imaging on a Jeol JEM 1400 transmission electron microscope (JEOL Peabody, MA). Phage head and tail measurements were obtained from a minimum of 10 representative phages from several prepared grids and analyzed in Image J. All values are reported as the mean ± SE of the mean (SEM). Descriptive statistics, including a two-tailed Student t-test, one way ANOA, were used to assess statistical significance using Excel (Microsoft, Redmond WA) and Minitab (Minitab, Inc. State College PA).

### Western blotting

Cultures were harvested at 6 days, centrifuged at 12,000 x g for 30 min and supernatants sterile filtered through a 0.22 μm filter. Samples were boiled in NuPAGE LDS sample buffer (Invitrogen, Carlsbad CA) and 5% BME for 5 min and resolved by SDS/PAGE on 4–12% NuPAGE Bis-Tris gels (Life Technologies), transferred to PVDF membranes and blocked in TBS containing 0.1% (weight/vol) Tween (TBS-T) and 5% (weight/vol) nonfat dry milk for 90 min at room temperature. Membranes were probed with monoclonal antibodies directed against heavy (TetE3, Abcam, Cambridge, MA) and light chains (Clone 604023 R&D Systems, Inc. Minneapolis, MN) of tetanus toxin at 1:1000 in TBS-T and 5% (weight/vol) BSA overnight at 4°C. Primary antibodies were visualized with AP-conjugated secondary antibodies (Sigma, St. Louis MO) at 1:20,000 dilution and colorimetric detection with nitro-blue tetrazolium chloride (NBT) AND 5-bromo-4-chloro-3'-indolyphosphate p-toluidine salt (BCIP).

## Results

**Fig 1:**
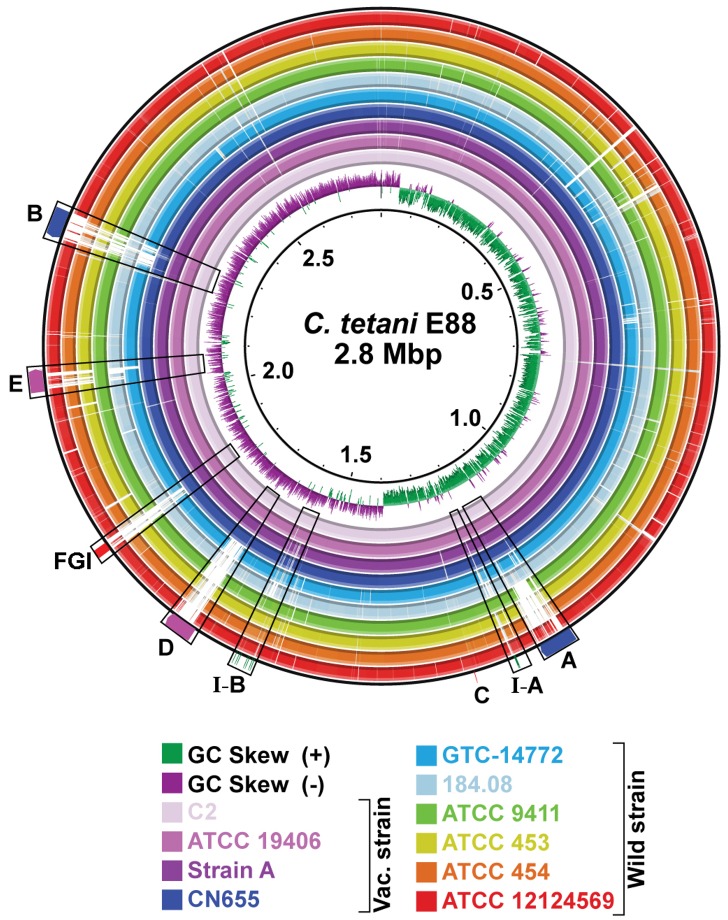
BLAST analysis of *C*. *tetani* draft genome sequences. *C*. *tetani* strain genomes were compared by pairwise BLAST using BRIG against Strain E88 (2.8 Mb, GenBank accession number NC_004557). Genomes shown include (from innermost to outermost ring) C2, ATCC 19406, Strain A, CN655, GTC-14772, 184.08, ATCC 9441, ATCC 453, ATCC 454, and 12124569 (See Methods for BioSample and nucleotide accession numbers). Genomic regions with < 80% nucleotide identity compared to reference sequence are indicated by white gaps. Central two rings (green and purple) represent GC skew, calculated for the E88 reference strain. Variable regions are labeled: prophage insertion regions A–E, CRISPR/Cas array I-A and I-B, and FGI.

Data from four newly sequenced strains (n = 3) were assembled by aligning against the genome sequence of Harvard strain E88 as a reference template [14]. Each genome was annotated and submitted to GenBank [31]. Comparative genomic analysis was conducted using these and 7 other previously sequenced strains (Table 1). Genetic variation among these strains was primarily from SNP content, five predicted prophage insertion sites (Fig 1A–1E), variations in three CRISPR/Cas arrays (I-A, I-B and III-A), and rearrangement and acquisition of polysaccharide modifying genes within a flagellar glycosylation island (FGI).

### Chromosome

#### SNPs

**Fig 2:**
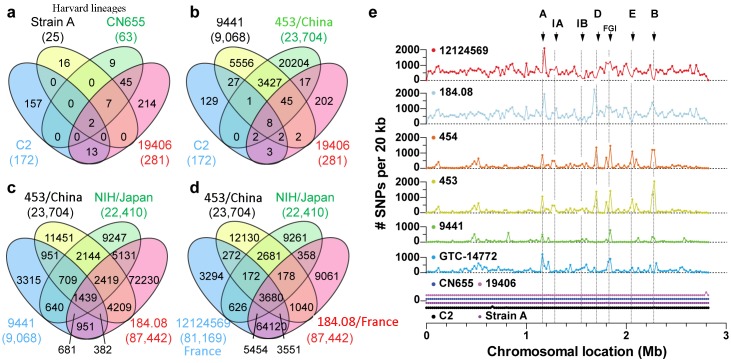
Distribution and frequency of SNPs in *C*. *tetani*. (A-D) Venn diagram of SNPs present in wild and vaccine strains of *C*. *tetani*. Strain E88 was used as a reference for determining SNP calls. Venn diagrams for strain sub-groups: (A) Harvard-derived vaccine strains C2, CN655 and Strain A [13], and ATCC 19406; very few SNPs were identified for all 4 strains, (B) Strains sequenced in the present study: C2, ATCC 19406, ATCC 9441, and ATCC 453, (C) and (D) Venn diagram of wild strains, ATCC 453, ATCC 9441, GTC-14772, 184.08 and 12124569. Strains 184.08 and 12124569. (E) Frequency distribution plot of SNP density (#SNPs/20 kb) and location along the E88 reference genome (chromosomal location). Regions displaying greater SNP frequency were associated with mobile genetic elements and indicated with arrows corresponding to regions identified in Fig 1: prophage insertions (A—E), CRISPR/Cas arrays (I-A and I-B), and FGI.

Chromosome sizes from the 11 strains were uniform at 2.80 ± 0.04 Mbp with GC content of 28.6 ± 0.1%. Harvard strains C2, Strain A, and CN 655 (Fig 1, represented by three inner BLAST rings) were nearly identical to strain E88 diverging only by 172, 25 and 63 SNPs, respectively (Fig 2). Surprisingly, strain ATCC 19406, which is not classified as a Harvard strain, was found to be identical to strain E88 aside from 281 SNPs in the chromosome. Strain ATCC 19406 does not carry a plasmid, however (see next section). Six wild-type strains had significantly more SNPs than the vaccine strains. ATCC 9441 is the most closely related wild-type strain to the Harvard strains having 9,068 SNPs when compared to the E88 reference strain (Fig 2b). The three East Asian strains (ATCC 453, ATCC 454, and GTC-14772) each have about 22,000 SNPs, while the strains isolated in France (184.08 and 12124569) have more than 81,000 SNPs (Fig 2a–2d).

Wild strains collected from similar geographic locations shared SNPs. For example, ATCC 453 and ATCC 454, both isolated in China [32, 33] shared 19,804 polymorphisms out of 23,704 (84%) and 22,392 (88%), respectively. Less overlap existed between the China strains and a strain recently isolated in Japan (GTC 14772), where about 28% of the SNPs were shared between the China strains. Similarly, strains 12124569 and 184.08 both isolated in France shared 76,805 SNPs out of 81,169 (95%) and 87,442 (88%), respectively (Fig 2d). Less overlap existed between the France and China isolates (7.9%–8.2%), France versus Japan (9.7%–10.6%) and France versus ATCC 9441 (3.7%–3.8%) (Fig 2c and 2d). Regions of greater SNP frequency had lower BLAST identity across all strains examined, and arose almost exclusively near mobile elements for flagellar glycosylation, predicted prophages, and CRISPR/Cas arrays (Fig 2e).

#### Flagellar glycosylation islands and bacterial swarming

**Fig 3:**
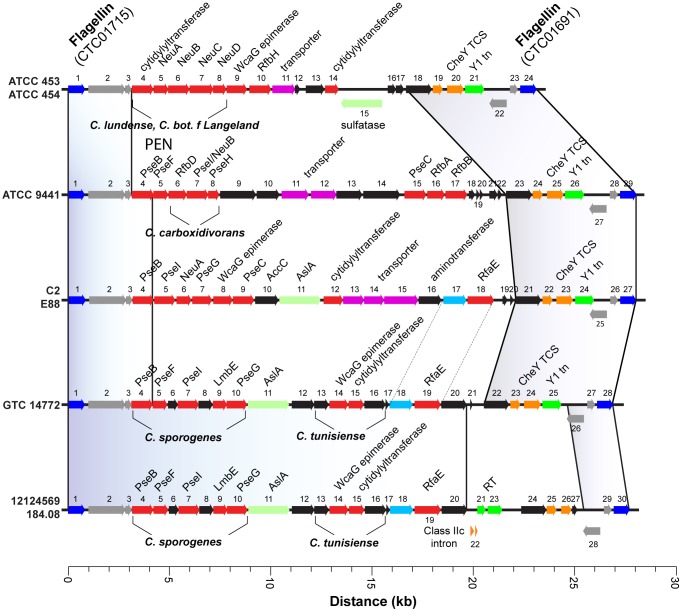
FGI is a mobile element in *C*. *tetani*. Annotation and alignment of the FGI genomic region across sequenced *C*. *tetani* strains. The FGI contains at least 80 predicted proteins that include the flagellar structural and polysaccharide processing enzymes in the sialic-acid like biosynthetic pathway (legionaminic acid and pseudaminic acid). Aligned regions that encompass *fla* flagellin genes (shown in blue, CTC01691—CTC01715) span 30 kb between 1,801,310–1,829,794 (*C*. *tetani* E88 reference). Shaded regions define the boundaries of highly conserved sequences across strains, e.g. *fla* CTC01691 –predicted protein adjacent to CheY for strains ATCC 453/454, ATCC 9441, C2/E88, and GTC-14772. See shaded region that are conserved between GTC-14772 and 12124569/184.08 (predicted proteins 1–20). Clusters of orthologous genes and moderate conservation with known FGI from the genus *Clostridium* (*C*. *botulinum*, *C*. *carboxyvidorans*, *C*. *lundense*, *C*. *sporogenes*, and *C*. *tunisiense*) are labelled. Predicted Y1 transposases (green) not conserved in French strains 184.08 and 12124569, contain a predicted group IIc intron (element 22) and reverse transcriptase (element 23). Predicted glycosylation enzymes (red), transporters (magenta), CheY two-component system (yellow), and proteins of unknown function (black) are shown.

SNP clustering was observed around a variable ~28kb region mapping to a gene locus of at least 80 predicted genes required for flagellar apparatus assembly and flagellar glycosylation (Figs 1 and 2e). Depending on the strain, an island of 23–29 predicted genes was found residing between mobile elements for reverse transcriptase and either a Y1 transposase or a group IIc intron (Fig 3). Most of these genes were predicted to participate in O-linked glycosylation of flagella [34–36]. About 18 genes within the FGI were organized along five distinct patterns depending on the *C*. *tetani* strain, and which in some cases were identical to known FGI gene arrangements found in other *Clostridia* (Fig 3). Overall, FGI organization was identical across all Harvard strains but showed striking uniqueness compared to wild type strains. The FGI loci in strains ATCC 453 and 454 were identical to each other and retained high synteny for five genes found in *C*. *ludense* and some *C*. *botulinum* strains for legionaminic acid biosynthesis. The locus in strains ATCC 453 and 454, however, lacked similarity to other *C*. *tetani* strains examined. Strains GTC 14772 (Japan), 184.08 (France) and 12124569 (France) maintained the best conserved FGI loci among themselves and compared to FGI loci found in *C*. *sporogenes* and *C*. *tunisiense* (Fig 3).

**Fig 4:**
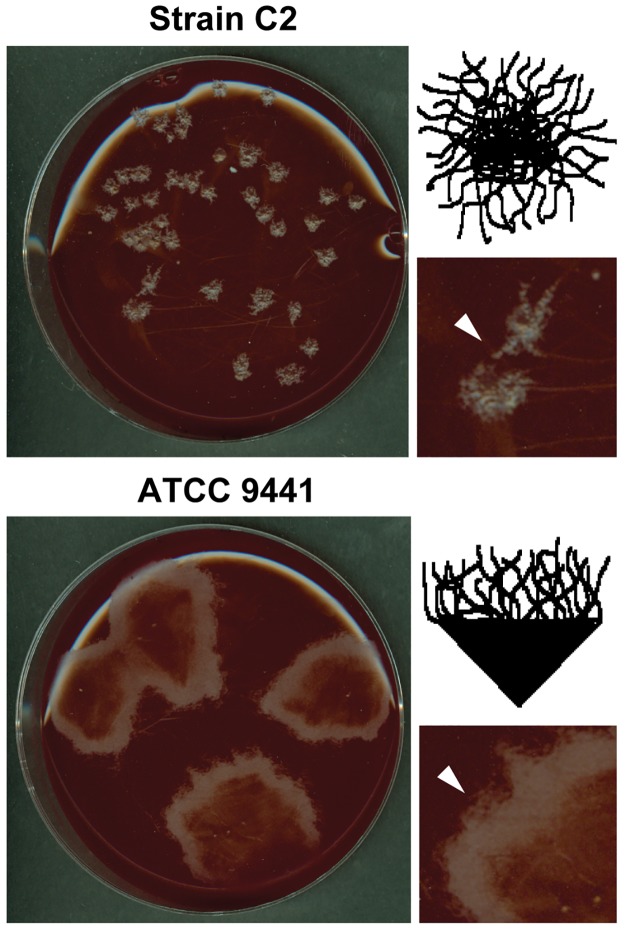
*C*. *tetani* motility on blood agar. Shown are representative examples of strain-specific motility and swarming phenotype in C2 and ATCC 9441 strains of *C*. *tetani*. Strains were plated onto blood agar plates and incubated for 48 hrs. Swarming behavior, exemplified by large spreading colonies is observed only in wild strain ATCC 9441. Strain C2 forms small, compact filamentous colonies (white arrow in magnified view). Strain ATCC 9441 forms large swarming colonies with hemolysis in the center, and a migrating boundary (white arrow in magnified view). Compare illustrations depicting filamentous form (top) to an irregular filiform margin (bottom).

ATCC 9441 was the only strain with an intact pseudaminic acid biosynthesis pathway, which predicts a high motility phenotype [35–39]. To test motility, we examined colony swarming by Harvard-derived isolates (C2 and ATCC 19406) and wild-type strains ATCC 9441, ATCC 453 and ATCC 454 on blood agar plates. Except for ATCC 9441, all strains exhibited minimal motility, and formed small (<5 mm) opaque rhizoid colonies within 24 h (Fig 4). A central site of alpha hemolysis can be, but is not always visible in these colonies. Strain ATCC 9441, in contrast, forms large, rapidly swarming halo-like colonies without distinct rhizoidal character and without a defined central mass. Irregular shaped halos have a defined filiform margin (Fig 4). These distinguishing patterns of colony formation among the strains does not prove the ATCC 9441 phenotype is due to flagellar modification with pseudaminic acid, but rapid spreading growth is consistent with this genotype in other fast swarming pathogenic bacteria [36, 38, 39].

#### Prophage and phage induction

**Fig 5:**
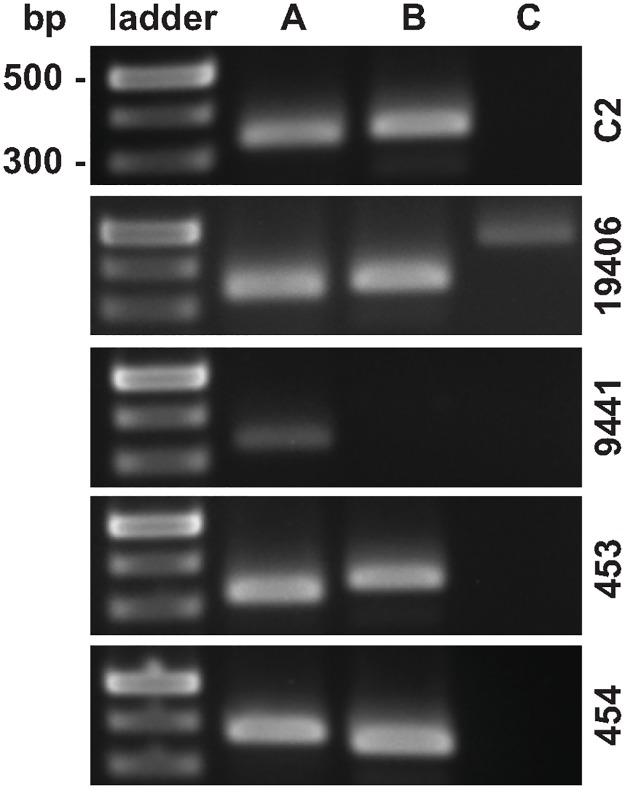
Phage typing *C*. *tetani* strains. Phage-specific inverse PCR of *C*. *tetani* phage particles. Phage particles were purified from cultures (60 sec UV irradiation and 24 hr incubation) and extensively treated with DNase to remove genomic and plasmid DNA contamination prior to PCR amplification assessed by the absence of RecA and tetX genes. Inverse PCR primers were specific to phage sequences flanking attL and attR attachment sites. Phages labelled A—C correspond to prophage integration sites A-C (Figs 1 and 2E).

Five distinct prophage integration sites were identified across all 11 genomes analyzed [26] (Figs 1 and 2e). We analyzed predicted phage proteins flanking the integration sites and identified 14 distinct phage-related serine recombinases (S1 Fig and S2 Appendix). All Harvard strains, including strain ATCC 19406, contain two distinct prophages lysogenized at sites A and B (Table 2). Prophages in each site are identical among the Harvard strains. A third prophage in insertion site C was identified in Harvard strain CN655 and ATCC 19406; this additional phage represented the highest source of genetic diversity among the vaccine strains. Insertion sites D and E did not carry full prophage genomes but harbored distinct phage-related genes consistent with remnants of earlier lysogenic infections. Nested PCR amplification targeting circular phage DNA determined that all three prophages A, B and C were inducible by UV irradiation from strains C2, ATCC 19406, ATCC 9441 and ATCC 453 (Fig 5). Neither PCR analysis nor DNA sequencing of isolated phage particles found evidence for viable prophage in sites D or E.

**Table 2:** Sequenced *Clostridium tetani* phage.

Strain	Integration Site	Accession	Size (bp)	GC (%)	# ORFs	Misc. features and notes
C2	A	KM983333	47,020	28.4	67 (39)	(+), SER, TERM3/6, SPP1
C2	B	KM983334	38,243	29.1	66(26)	(-), SER, TERM1, HK97
19406	A	KM983330	47,020	28.4	67 (39)	(+), SER, TERM3/6, SPP1
19406	B	KM983331	38,243	29.1	66 (26)	(-), SER, TERM1, HK97
19406	C	KM983332	35,703	29.2	63 (33)	(-), SER, TERM1, HK97
9441	A	KM983329	46,895	28.7	78 (37)	(+) SER, TERM6, SPP1
453	A	KM983327	42,158	29.5	62 (26)	(+), SER, TERM 3/6, SPP1
453	B	KM983328	36,712	29.1	61 (25)	(-), SER, TERM1, HK97

Among the wild-type strains, 11 intact prophage genomes were distinct from the three prophages in Harvard vaccine strains. Similar to the Harvard strains, regions D and E in wild-type strains encoded a variety of phage related genes but appeared to harbor incomplete prophages. For wild type strains ATCC 453 and 454 (both from China), respective prophages in sites A and B were identical to the A and B prophages among the Harvard strains but were not present in strains isolated in France or Japan. Strain ATCC 9441 was positive for prophage in integration site A, but carried no prophages in regions B or C. Phylogenic analysis of four critical phage genes (serine recombinase, portal protein, terminase, and tail tape measure protein) was performed for all 14 identified prophages (S1 and S2 Figs) and shows that all are commonly found among soil and gut-borne bacteria (S3 Fig and S1 Table).

**Fig 6:**
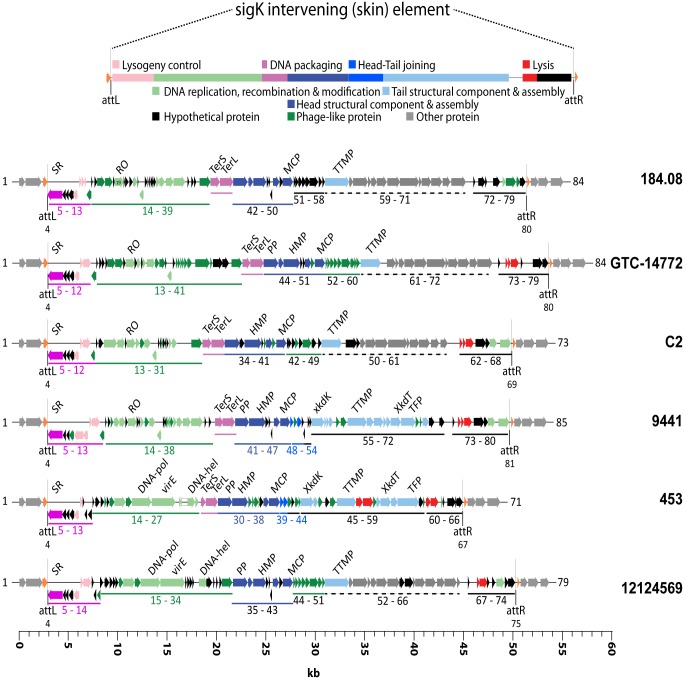
Genomic organization of sigK intervening element in *C*. *tetani*. Shown is a schematic representation of the genetic organization of prophage genomes identified in all *C*. *tetani* strains at insertion site A (Fig 1). The identified prophage resembles a *Bacillus subtilis* phage DNA-like sigK intervening (skin) element. Predicted ORFs are represented as arrows in their respective orientation covering chromosomal location 1,135,168–1,192,484 nt in E88 reference strain. Conserved 5’- and 3’-flanking sequences (light grey), phage attachment sites attL and attR including the disrupted sporulation sigma factor, sigK (orange) are shown. Functional modules were assigned based on annotation and genomic organization for DNA packaging (magneta), capsid morphogenesis (blue), tail morphogenesis (light blue), lysis (red), lysogeny (pink), and DNA replication, transcription, and gene regulation (light green). Lysogenic phages were most highly conserved in genes associated with lysogeny and phage replication. Phage like-proteins (green) and hypothetical proteins (black) are shown as well. Predicted head-tail joining proteins (blue) and phage tail proteins (light blue) could only be identified in ATCC 453 (regions 39, 40 and 45–59) and ATCC 9441 (regions 48–50 and 55–72). Abbreviations: attachment sites **attL** and **attR**; **XkdT**, baseplate protein; **DNA-h**, DNA helicase; **DNA-pol**, DNA polymerase; **HMP**, head morphogenesis protein; **MCP**, major capsid protein; **PP**, portal protein; **RO**, replisome organizer; **SR**, serine recombinase; **TFP**, tail fiber/collar protein; **XkdK**, tail sheath protein; **TTMP**, tail tape measure protein; **TerS** and **TerL**, terminase small and large subunit; **virE**, virulence factor E.

From all strains examined, prophage elements from regions A-C can be organized into 7 predicted phage genomes with distinct loci for different stages of infection: (a) DNA replication, recombination, and modification (b) DNA packaging (c) Head structural component and assembly (d) Head-tail joining (e) Tail structural component and assembly (f) Lysis, and (g) Lysogeny control (Fig 6). Prophage sites A-C were flanked on 5’- and 3’- ends by phage attachment sites (attP) and a conserved serine recombinase [40]. Regions D and E were flanked on the 5’ end by a serine recombinase but on the 3’ end by XerC/XerD tyrosine integrase [41].

Prophage within insertion site A always resided within a sporulation-specific alternative sigma factor K (*sig*^*K*^) gene locus, which resembles the DNA-like *sig*^*K*^ intervening (skin) element found in *Bacillus subtilis* [42], *C*. *difficile* [43], and *C*. *perfringens* [44] (Fig 6). All prophages found at site A share greater conservation than prophages found at the other insertion sites with highest conservation near the 5’- and 3’- insertion sites. Most divergence in this case emerged in various proteins required for phage attachment, DNA packaging, and tail assembly (S2 Table). Gene organization among prophages site A, specifically in the tail assembly proteins, e.g. Xkd genes XkdK—T, suggest they are from the Myoviridae family. Prophages in region B are likely to be in the Siphoviridae family. High homology across prophages in site A seems to reflect the importance of *sig*^*K*^ in promoting sporulation [43, 44].

**Fig 7:**
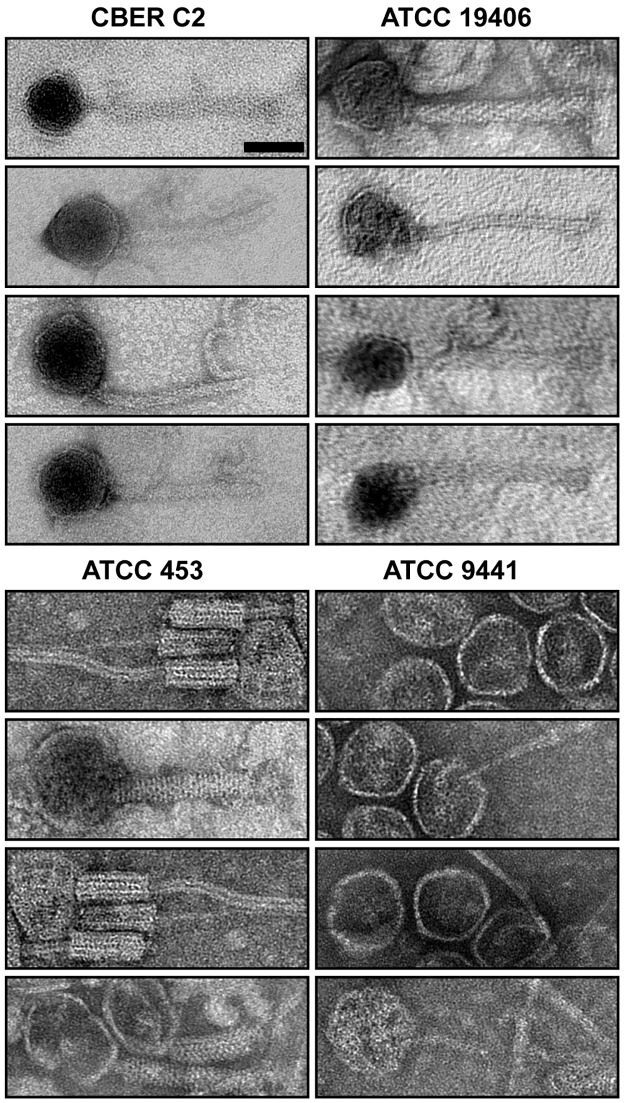
Electron microscopy of bacteriophages isolated from *C*. *tetani*. Representative transmission electron microscopic (TEM) images of UV-induced bacteriophages isolated from *C*. *tetani* strains C2, ATCC 19406, ATCC 453, and ATCC 9441. Distinct morphological features are evident in isolated bacteriophage, e.g. contractile tail for ATCC 453 and long, flexible tails for ATCC 19406. Note the presence of striations of phage tails observed for ATCC 453. Empty phage heads, lacking tails were observed in phage isolates from ATCC 9441. Scale bar = 50 nm. See S1 Appendix for detailed measurements.

Phage particles obtained from strains C2, ATCC 19406, ATCC 9441, ATCC 453 and ATCC 454 were visualized by EM (Fig 7). In spite of chromosome sequence data predicting up to five prophages per strain, we found one morphology per strain by EM and only three distinct phage morphologies overall. Structural analyses of the images could not link phage to regions A, B or C. Detailed physical measurements of each phage are provided in supplemental results (S1 Appendix).

#### Conservation of CRIPSR/Cas system

**Fig 8:**
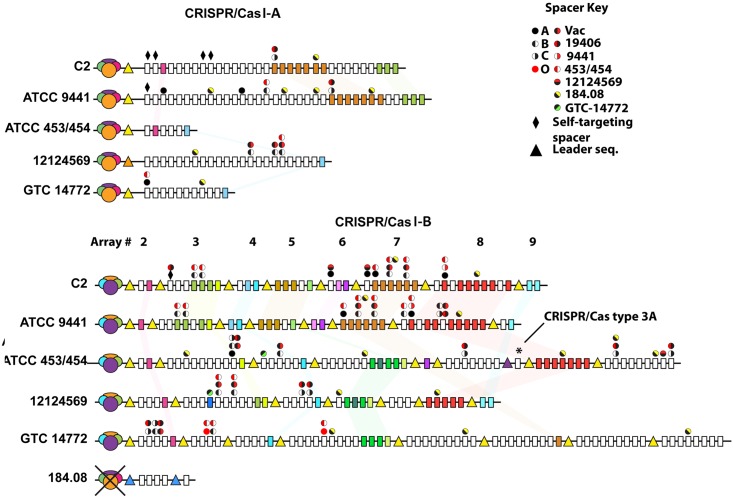
CRIPSR spacer diversity and conservation. CRISPR/Cas arrays are organized into two primary arrays, I-A (A) and I-B (B) (see Figs 1 and 2) with low homology across all 11 sequenced strains. CRISPR/Cas arrays were organized into a single large array (I-A, array 1) or shorter arrays (I-B, arrays 2–9) consisting of a conserved leader sequence (triangle) and repeating alternating units of linkers and spacers (rectangles), color-coded based on conservation across *C*. *tetani* strains. Self-targeting spacers present in strains C2 and ATCC 9441 are shown (diamond) as well as spacers targeting identified *C*. *tetani* phages. CRISPR/Cas arrays present in vaccine strain C2 and ATCC 9441 were most similar among the oldest spacers at the tailing ends of both arrays. CRISPR/Cas spacers in GTC-14772 were least similar to other *C*. *tetani* strains and included an additional array that mapped to a 141kb contig with an incomplete complement of CRISPR/Cas proteins and phage-like proteins. CRISPR/Cas proteins were immediately upstream of the leader sequence for I-A, and distributed throughout the array for I-B. A CRISPR/Cas type III-A array was identified upstream of a single array (asterisk) in ATCC 453/454. A functional set of CRISPR/Cas proteins was absent in strain 184.08 despite the presence of 5 spacers and a distinct leader sequence. See S3 Table for CRISPR/Cas proteins.

Because of the number and diversity of predicted phage genomes, we evaluated the CRISPR/Cas systems for sequence conservation and organization across *C*. *tetani* strains. Two major CRISPR/CAS systems, I-A and I-B were found in 10 strains with a smaller III-A found in two strains (Fig 8, S3 Table, S2 Appendix) [45, 46]. All three CRISPR/Cas systems exist downstream of transposases rendering them subject to re-organization from either horizontal transfer or deletion cross-over events.

CRISPR/Cas system I-A was located between prophage insertion sites A and C (Fig 1). The I-A system was the simplest complete CRISPR/Cas loci in the genomes, with defensive spacer sequences against a different number of previous phage exposures which varied depending on the strain: six spacers for strains ATCC 453 and 454 (China), 11 spacers in GTC 14772 (Japan) and 23 spacers in strain 12124569 (France) (Fig 8). Strain 184.08 (France) was unique in that it did not have a CRISPR I-A system due to a 12kb deletion [16]. The I-A CRISPR/Cas system in strain ATCC 9441 had the highest homology with Harvard strains sharing 10 (31%) of the phage-related spacers while no CRISPR/Cas spacer overlap was observed between Harvard strain E88 and other wild-type strains.

CRISPR/Cas system I-B was located near prophage insertion site D. Unlike I-A, the I-B system was composed of seven to nine interspersed smaller arrays of phage-related spacers (Fig 8). Two distinguishing qualities stand out in the I-B array. Although I-B in strain GTC 14772 (Japan) is the longest having 62 spacers, only three spacers were conserved with strains ATCC 453 and 454 (China) and strain 12124569 (France) suggesting the Japan strain diverged from other strains long ago. The I-B loci in wild-type ATCC 9441 and the Harvard strains, on the other hand, were nearly identical (72% by spacer homology) across all elements of 39 phage-related spacers (Fig 8). This inter-relatedness of I-B content was highest between Harvard and strain ATCC 9441 compared to all other wild type strain (S4 Fig).

Self-targeting CRISPR/Cas spacers can facilitate an autoimmune-like genetic control of specific genes of the host organism [47]. The Harvard strains contain five self-targeting spacers while ATCC 9441, has one such spacer (S3 and S4 Tables). Two of these spacers targeted sporulation sigma-E factor processing and stage IV sporulation protein A spoIVA, which might contribute to Harvard strains being impaired at spore formation compared to wild-type strains [13, 48].

### Plasmid and TetX

**Fig 9:**
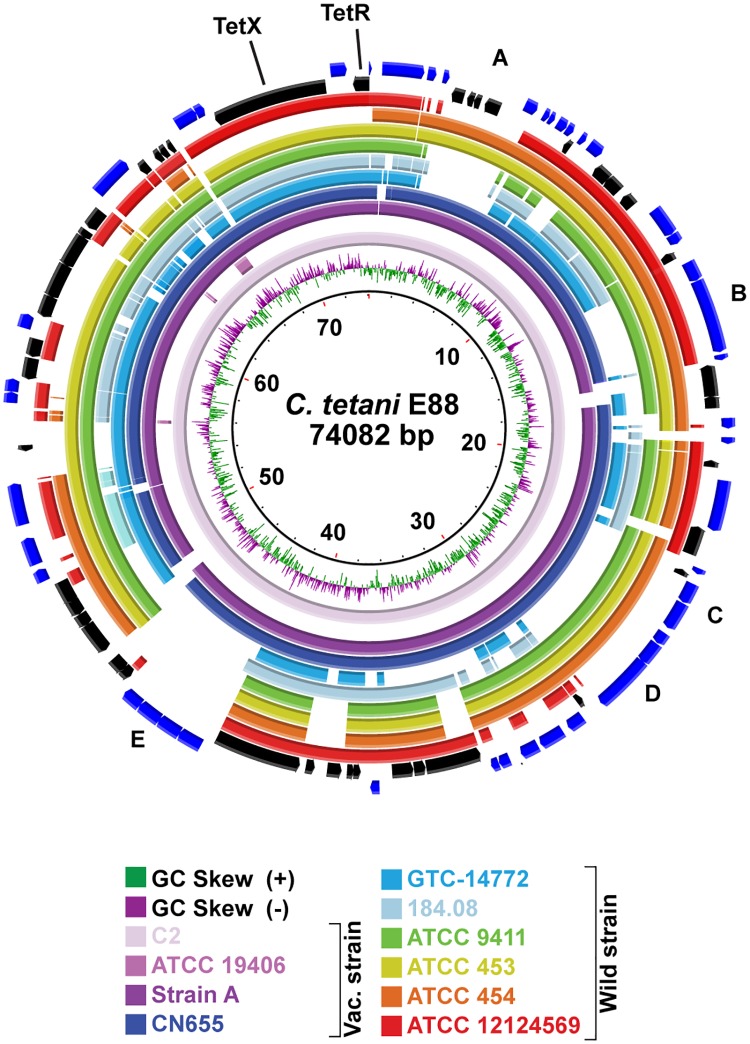
BLAST analysis of *C*. *tetani* plasmid sequences. *C*. *tetani* plasmids were compared against pE88 (74 kb, GenBank accession number NC_004565). Outermost two rings show predicted gene products for (+) and (-) strand. Position of tetanus toxin regulator (*TetR*) and toxin (*TetX*) genes are shown. Regions of nucleotide identity <80% are indicated by white gaps. *C*. *tetani* strain ATCC 19406 lacks the plasmid and ATCC 454 is missing both *tetR* and *tetX* genes. Regions A-E represent deletions in the plasmids from WT strains: uviB and DNA directed RNA polymerase sigma-70 factor (A), ftsX/ABC anti-microbial transporter system (B), OmpR/BaeS two-component system (C), ftsX/ABC-type lipoprotein export system/permease complex (D), and ABC multidrug-resistance transporter/permease complex (E).

Sequence variations in the toxin-encoded plasmid were minor among Harvard strains except for ATCC 19406, which despite very high chromosomal homology with Harvard strains, was missing the plasmid rendering it non-toxigenic (Fig 9 and Table 1). All wild-type strains harbored a single plasmid but with much divergence from pE88 (Fig 9). Plasmid GC content across the strains averaged 24.6 ± 0.3%, lower than 28–29% observed in the chromosome. Of the four plasmid-bearing Harvard strains, plasmid size was 72.0 ± 1.8 kb whereas it was 66.3 ± 19.2 kb among wild type strains. Plasmid from strain ATCC 454, a clinical strain isolated from human gut flora in China in the 1920s, had >95% sequence identity with E88 (Fig 9, orange ring) but was non-toxigenic due to a 20 kb deletion encompassing TetR and TetX genes suggesting the neurotoxin is not required for intestinal colonization. Plasmid from strain ATCC 453, another clinical strain from China did not have this deletion and was otherwise identical to ATCC 454. ATCC 454 was the only wild-type strain lacking TetR and TetX genes (Fig 1b, 1A–1E).

Toxin gene, TetX, was identical across all Harvard strains and was conserved with 99.3–99.4% identity across wild type strains, encoding a predicted protein of 1315 amino acids in all cases. Strains 184.08 and 12124569 isolated in France had 26–29 SNPs, strains ATCC 453 and 454 isolated in China had 23 SNPs and the Japan strain had 19 SNPs compared to strain E88. TetX from strain ATCC 9441 was most closely related to the Harvard strain, having only 4 SNPs, three of which produced amino acid substitutions. The TetX regulator, TetR, was 100% identical across all vaccine and wild type strains carrying this gene.

## Discussion

We have sequenced the genomes of four *C*. *tetani* strains: ATCC 19406, ATCC 453, ATCC 9441 and Harvard vaccine strain C2. Data was compiled from triplicate sequencing runs. Genetic analysis was performed on these and seven other *C*. *tetani* genome sequences available in GenBank, which are: three Harvard strains (E88, CN655 and Strain A) and five clinical strains isolated from France, China or Japan [15, 16]. The genome from strain E88 was used as reference. Our results add to a recently published study comparing three vaccine and two wild-type strains by Bruggemann et. al. [2015]. Genetic identity among the 11 strains was high. All five Harvard strains, for example, have 25 to 281 SNPs from a genome of 2.8 Mbp in spite of significant passaging over 60+ years for research and commercial use. Our analysis along with data from Bruggemann et. al. [2015] demonstrates Harvard strains are a clade rather than a single strain. The European Harvard strains CN655 and Stain A have fewer SNPs, 63 and 25, respectively, compared to the North American strain C2 having 172 SNPs relative to strain E88. All vaccine strains share non-sporulating and high toxin producing phenotypes suitable for toxoid manufacture.

Strain ATCC 19406 was found to be non-toxigenic yet nearly identical to all Harvard strains having 281 SNPs relative to the E88 genome. We believe strain ATCC 19406 was likely isolated around 1919 in the US Hygienic Laboratory which had isolated the Harvard progenitor strain two years earlier [9–13]. Lacking the TetX toxin gene, we presume strain ATCC 19406 has been passaged far less than Harvard production strains.

SNP analysis using ATCC 19406 as the reference strain, with SNPs in parentheses shows: ATCC 19406 (0), strain CN655 (9), Strain A (21), strain C2 (157), and strain E88 (281). Strain E88 showed the greatest SNP content consistent with this strain having been sent to the UK in the 1920s [11, 12]. Strain C2, the North American vaccine strain, was derived from the USA Type II strain collected around 1918 [10–12], and has 157 SNPs compared to strain ATCC 19406 consistent with strains C2 and E88 having been used extensively for research and product manufacture. The strain C2 preparation sequenced in our study had been stored lyophilized since 1965 and had been passaged one time between 1962 and 1965. The 157 SNPs in strain C2 compared to ATCC 19406 equates to a mutation rate of 1.3 x 10^−6^ substitutions per base pair per year assuming 1919 to 1962 as the years separating these strains. Using the same mutation rate to calculate divergence between strain ATCC 19406 and European Harvard strain E88 predicts 77 years separate these strains (1919 + 77 = 1996), which is coincident with strain E88 being sequenced around 2003 [14]. The derived mutation rate for the vaccine strains is typical for pathogenic bacteria but is unusually high for spore-formers that can exist in a spore state for protracted periods [49]. Mutation rate(s) for wild type strains could not be estimated from the available data.

Bacteriophage exposure history (CRISPR-Cas systems) and current prophage insertion elements are nearly identical among all Harvard vaccine strains but quite diverse among the wild-type strains. Harvard strain CN655 is the only strain that shares an identical prophage in insertion site C with strain ATCC 19406, which along with only 9 SNPs makes these two strains the most closely related among the vaccine strains. All *C*. *tetani* strains share five defined phage insertion sites. Phage phylogenic analysis indicates the 14 prophages identified are common in soil and gut-borne bacteria (S1, S2 and S3 Figs). Prophages within insertion site A universally reside in a *sig*^*K*^ gene locus, which resembles the DNA-like *sig*^*K*^ intervening element found in *Bacillus subtilis* [42], *C*. *difficile* [43], and *C*. *perfringens* [44]. Since our vaccine strain does not form spores, it is tempting to attribute spore deficiency to prophage disruption of sig^K^, however, this is not likely the case because wild type spore-forming strains have similar prophage insertions and strains ATCC 9441 and ATCC 453 readily form spores in contrast to Harvard strain C2 and ATCC 19406. Deficient sporulation among Harvard strains can more likely be explained by two self-targeting spacers found in Harvard CRISPR array I-A, which target sporulation sigma-E factor and Stage IV sporulation protein A. These spacers are absent from wild type strains.

Flagellar glycosylation patterns can be representative of phenotypic and pathogenic qualities among many bacterial species and may contribute to immune evasion [34, 38]. Flanking regions of the FGI found in *C*. *tetani* are susceptible to the highest rates of SNP substitutions compared to other mobile elements examined in this study. Gene arrangement within the FGI was well conserved in certain instances. For example, all Harvard strain FGI loci were identical, but the loci did not resemble the FGI found in wild-type strains. Strains ATCC 453 and 454, both isolated in China, share identical FGI loci, much of which are conserved in the FGI found in *C*. *lundense* and some strains of *C*. *botulinum* [34]. Strain GTC 14772 (Japan) and strains 184.08 and 12124569 (both isolated in France) have nearly identical FGI loci, yet these have no synteny with other *C*. *tetani* FGI. Strains ATCC 453 and 454 lack *Pse* genes required for pseudaminic acid biosynthesis, but these genes were present in various arrangements in the other nine *C*. *tetani* strains evaluated (Fig 3). Strain ATCC 9441 was the only strain that retained a complete set of genes required for pseudaminic acid biosynthesis, which is a genotype associated with rapid motility and greater pathogenicity in other bacteria [36, 38]. We observed that strain ATCC 9441 exhibits greater motility and unique colony morphology on agar plates compared to our Harvard strain C2, ATCC 19406 and ATCC 453. Interestingly, FGI gene organization in all Harvard strains did not overlap with FGI loci from wild-type *C*. *tetani* strains or with FGI loci from other *Clostridia*. None of the *C*. *tetani* wild type strains exhibited synteny with the FGI from *C*. *difficile* [35].

The closest related wild-type strain to the Harvard clade is strain ATCC 9441, which shares less than 1% of its 9068 SNPs with the Harvard strains but retains good synteny within the CRISPR I-A array and has a nearly identical CRISPR I-B array found in Harvard strains. Among the wild type strains, the toxin gene TetX has the smallest number of SNPs in ATCC 9441. Strain ATCC 9441 also shares an identical prophage with the Harvard strains in insertion site A. As the Harvard strain originated in North America, genetic analysis demonstrates ATCC 9441 and the Harvard strains have a shared history of phage exposure and, therefore, may be derived from a common ancestor.

Aside from CRISPR/Cas homology to Harvard strains, comparisons of SNPs in wild-type strains suggest ATCC 9441 is inter-related to the other five wild type strains sharing about 38% of its SNPs with China strains ATCC 453 and ATCC 454, 24% SNP overlap with Japan strain GTC14772 and about 38% retention with both France strains (Fig 2C and 2D). Although SNP content can suggest a pattern of strain migration across large geographical distances, additional genomic data from more diverse strains is required to link genotypes to geographic points of origin.

Our primary focus in this study was to determine the extent of genetic diversity between our vaccine strain C2 compared to previously sequenced Harvard strains. The high level of genomic identity found in this study is consistent with *C*. *tetani* like, *C*. *botulinum* and *C*. *sporogenese* having remarkably stable genomes [50]. No pedigree was available for ATCC 9441 and only modest information was available for ATCC 19406, but based on sequence data, ATCC 19406 was derived from the original Harvard progenitor strain, whereas wild type strain ATCC 9441 appears related to the vaccine strains based on CRISPR/Cas homology and having the smallest number of SNPs in the TetX gene. Combined genomic evidence primarily from SNP overlap and CRISPR/Cas homologies provide tentative evidence that the 11 strains examined in this study are genetically linked across three continents but diversity among these elements including strain-specific alterations to FGI organization precludes an immediate explanation for how strains may have spread. In spite of sometimes extensive variations in parts of the genomes, all strains retained 100% homology in the tetanus toxin gene regulator, TetR, and >99% identity in TetX, indicating that unlike the toxin loci among *C*. *botulinum*, TetX is not subjected to extensive mutation or rearrangement [4, 7, 51–53]. For supporting vaccine manufacture, the absence of mutations in TetR or TetX provides assurance that all tetanus toxoid products made using any strain from the Harvard clade will likely be identical in terms of toxin antigenic content, reducing concern about genetic diversity adversely impacting vaccine quality.

## Supporting information

S1 Fig Phylogenetic tree: Serine recombinase. (A) Circular phylogenetic tree of predicted prophage serine recombinase genes. Multiple sequence alignment was performed using the MUSCLE algorithm with neighbor joining algorithm and phylogenetic trees were constructed by maximum likelihood analysis with bootstrapping (values <50 are shown in red). 90 protein accession numbers are color coded based on type of environmental isolate or species: *C*. *botulinum* (11, purple), *C*. *perfringens* (7, orange), *C*. *tetani* (14, Blue), soil (7, light brown), fecal-oral (26, green), waste water runoff (4, silver), and thermophilic organisms (21, red). Five distinct families of serine recombinase genes clustered based on insertion site (Φ A– Φ E). Predicted serine recombinase genes unassigned to an integration site are annotated with an X. (B) Percent identity matrix of predicted *C*. *tetani* serine recombinase genes where % amino acid identity is color-coded from blue (low sequence identity) to red (high sequence identity).
(TIF)

S2 Fig Phylogenetic analysis of predicted portal protein and terminase genes. Percent amino acid identity matrices for predicted *C*. *tetani* Portal Protein (A) and terminase TerL genes (B) and Tail Tape Measure Protein (C). Identity is color-coded from blue (low sequence identity) to red (high sequence identity). Multiple sequence alignment was performed using the MUSCLE algorithm with neighbor joining algorithm and phylogenetic trees were constructed by maximum likelihood analysis with bootstrapping (values <50 are shown in red). (A) 147 portal protein accession numbers were used in the analysis. Portal proteins were clustered into broad SPP1-, HK97-, and HK97/H-NS like portal protein families. The type of environmental isolate and bacterial species closely related to *C*. *tetani* portal protein is similar to what was found for serine recombinases: *C*. *botulinum* (22), *C*. *perfringens* (4), *C*. *tetani* (17), soil (25), fecal-oral (39), waste water runoff (12), and thermophilic organisms (28). Frame B: For terminase genes, 139 terminase accession numbers were used in the analysis. The type of environmental isolate and species are: *C*. *botulinum* (33), *C*. *perfringens* (2), *C*. *tetani* (14), soil (21), fecal-oral (43), waste water runoff (21), and thermophilic organisms (15). Low AA identity is seen for the majority of *C*. *tetani* TerL proteins.
(TIF)

S3 Fig 16sRR phylogenetic tree of bacterial species. (A) Circular phylogenetic tree of 16sRR constructed from 144 bacterial species (417 phage and 22 CRISPR/Cas proteins). Multiple sequence alignment was performed using the MUSCLE algorithm with neighbor joining algorithm and phylogenetic trees were constructed by maximum likelihood analysis with bootstrapping (values <50 are in red). Bacterial species are color-coded based on type of environmental isolate or species: *C*. *botulinum* (16, magenta), *C*. *perfringens* (1, orange), *C*. *tetani* (1, blue), soil and sediment (19, light brown), fecal-oral (62, green), waste water and runoff (23, silver), and thermophilic organisms (21, red). The *C*. *botulinum* strains were clustered along the tree into Group I, Group II, and Group III families with the exception of several distantly related BoNT-expression organisms, *C*. *argentinense* (BoNT/G) and *C*. *baratii* (BoNT/F). *C*. *tetani* showed significant similarity to sequenced strains *C*. *tetanomorphum* and *C*. *lundense* (*C*. *cochlearium* JCM 1396, despite having higher 16sRR sequence identity, was not present as the genome has not been sequenced). (B) Distribution of 16sRR sequences based on environmental isolate (fecal-oral, waste-water and runoff, soil and sediment) or species (*C*. *botulinum*, *C*. *difficile*, *C*. *perfringens*). Of the 144 bacterial species identified with high overall conservation in phage and CRISPR proteins, 43% were bacterial organisms predominately found inhabiting the gastrointestinal tract and feces. Redundant 16sRR sequences and species names were removed to better show representation. See supplemental S3 Table for sequence and accession number.
(TIF)

S4 Fig CRIPSR spacer conservation and conservation. CRISPR/Cas arrays are conserved in *C*. *tetani* strains. CRISPR/Cas arrays were identified by conserved leader and linker sequences, and aligned against E88 reference genome. Conservation is shown as rank (1 = high conservation and 40 = low conservation) calculated by the number of conserved spacer sequences per total # spacers for each large array, I-A and I-B and Type III-A (in ATCC 453 and ATCC 454). CBER C2 is similar to ATCC 19406, CN655 and Strain A. Mean rank score were C2 (13.4), ATCC 9441 (13.2), ATCC 453 (26.60), 12124569 (20.9), and GTC 14772 (31.9).
(TIF)

S1 Table Accession numbers for phylogenetic trees (16sRR and protein). Sequences corresponding to 16sRR were collated from 145 bacterial species (including *C*. *tetani* E88 reference strain) and categorized based on type of environmental isolate or species as in S3 Fig. When possible, the accession number for the noncoding RNA is given. For identified 16sRRs extracted from genomic sequences, the accession number and nucleotide position is given. 16sRR sequences for Group I–III *C*. *botulinum* sequences are given. Phage protein accession numbers (417) used for construction of phylogenetic trees corresponding to serine recombinase, portal protein, TerL, tail-tape measure protein are given (S1 and S2 Figs). Phage insertion sites (A-E) are listed for predicted phage proteins in *C*. *tetani* strains. Eight phage proteins identified in *C*. *tetani* strain GTC-14772 (6) and ATCC 453 (2) did not have accession numbers. Predicted proteins sequences for 22 CRISPR/Cas proteins with sequence identity to type IA, IB, and III systems.
(XLSX)

S2 Table Prophage accession information for compiling phage phylogeny. (XLSX)

S3 Table CRISPR linker sequences for vaccine and wild type strains. (DOCX)

S4 Table CRISPR linker sequences. CRISPR/Cas arrays were analyzed by CRISPR finder (http://crispr.u-psud.fr/). Shown are repeat sequences for CRISPRs identified in *C*. *tetani* strains. The number of actual CRISPR arrays in each is likely lower because the majority of genome information is unfinished WGS data. For all strains with the exception of 184.08, ATCC 453/454, and GTC-14772, two primary arrays exist having unique spacers (SP). A defective CRISPR/Cas system identified in strain 184.08, contained few spacer sequences.
(XLSX)

S5 Table PCR primers for prophages found in insertion sites A, B and C. (DOC)

S1 Appendix Bacteriophage/Prophage description. (DOC)

S2 Appendix CRISPR-Cas genetic conservation. (DOC)
