# Supplementary figures and images for: Comparative pathogenomics of Clostridium tetani
Source: PLoS One. 2017 Aug 11;12(8):e0182909. doi: 10.1371/journal.pone.0182909 (PMC5553647; doi:10.1371/journal.pone.0182909)

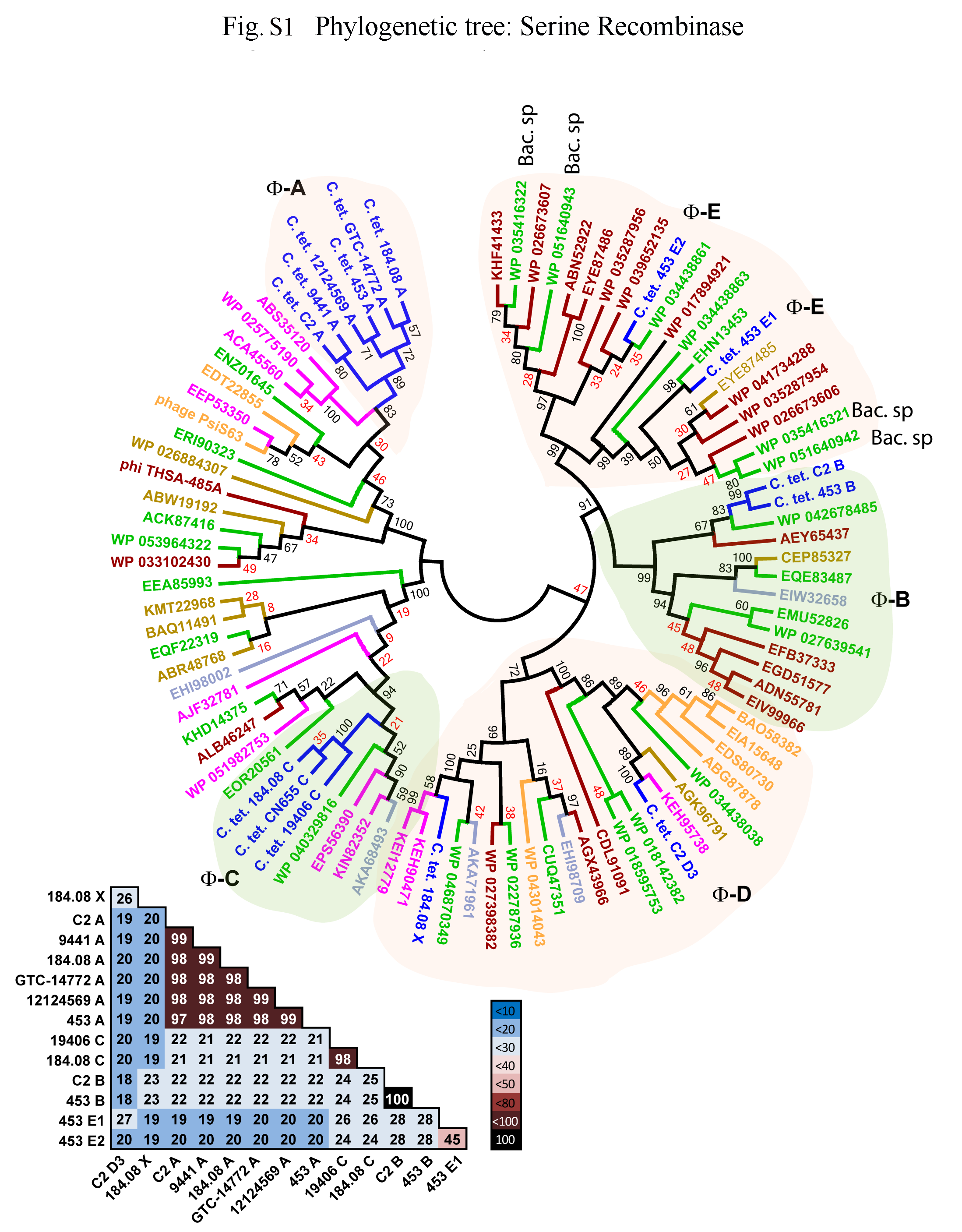

Supplement: S1 Fig — (A) Circular phylogenetic tree of predicted prophage serine recombinase genes. Multiple sequence alignment was performed using the MUSCLE algorithm with neighbor joining algorithm and phylogenetic trees were constructed by maximum likelihood analysis with bootstrapping (values <50 are shown in red). 90 protein accession numbers are color coded based on type of environmental isolate or species: C. botulinum (11, purple), C. perfringens (7, orange), C. tetani (14, Blue), soil (7, light brown), fecal-oral (26, green), waste water runoff (4, silver), and thermophilic organisms (21, red). Five distinct families of serine recombinase genes clustered based on insertion site (Φ A– Φ E). Predicted serine recombinase genes unassigned to an integration site are annotated with an X. (B) Percent identity matrix of predicted C. tetani serine recombinase genes where % amino acid identity is color-coded from blue (low sequence identity) to red (high sequence identity). (TIF) [file pone.0182909.s001.tif]

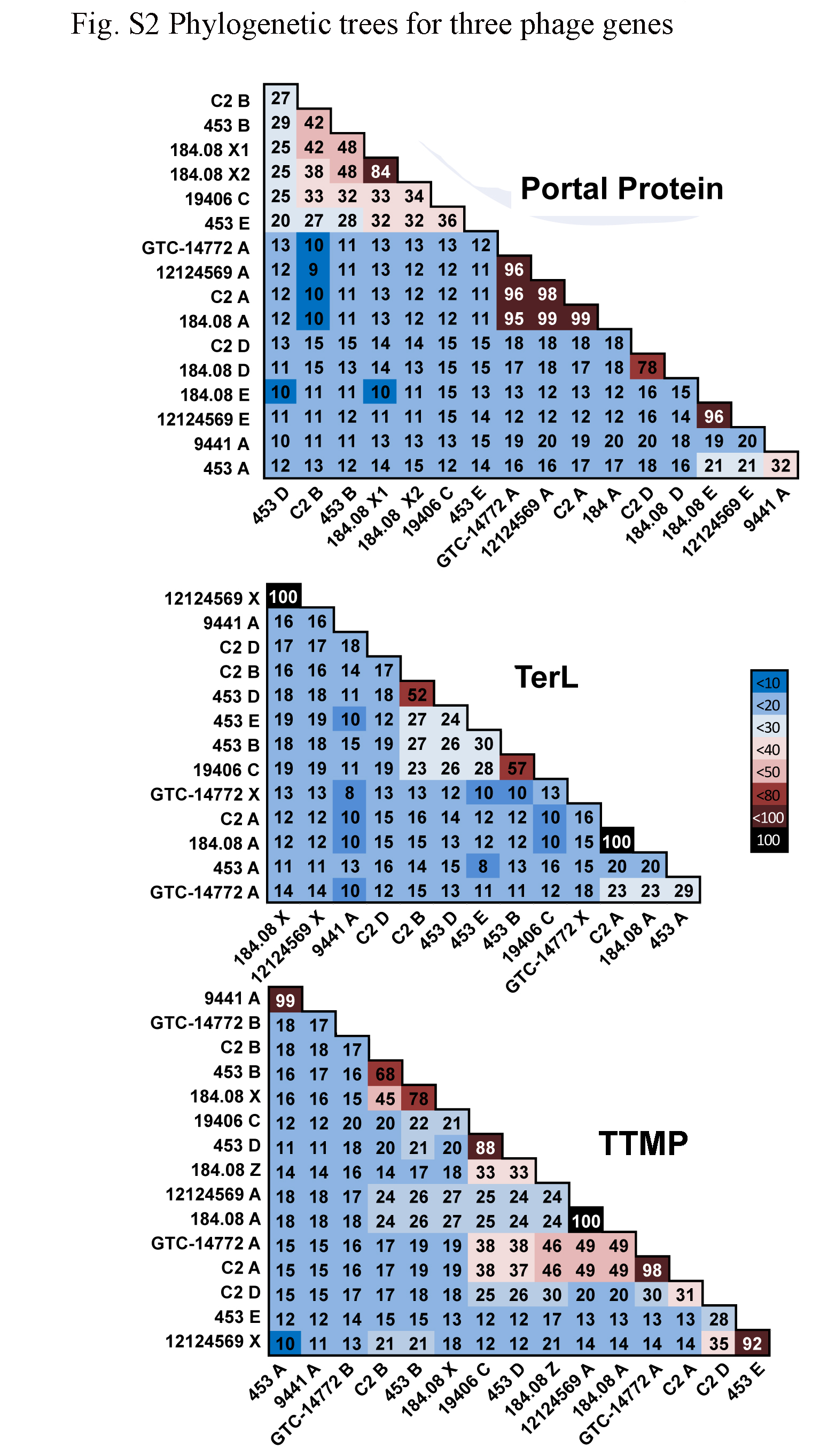

Supplement: S2 Fig — Percent amino acid identity matrices for predicted C. tetani Portal Protein (A) and terminase TerL genes (B) and Tail Tape Measure Protein (C). Identity is color-coded from blue (low sequence identity) to red (high sequence identity). Multiple sequence alignment was performed using the MUSCLE algorithm with neighbor joining algorithm and phylogenetic trees were constructed by maximum likelihood analysis with bootstrapping (values <50 are shown in red). (A) 147 portal protein accession numbers were used in the analysis. Portal proteins were clustered into broad SPP1-, HK97-, and HK97/H-NS like portal protein families. The type of environmental isolate and bacterial species closely related to C. tetani portal protein is similar to what was found for serine recombinases: C. botulinum (22), C. perfringens (4), C. tetani (17), soil (25), fecal-oral (39), waste water runoff (12), and thermophilic organisms (28). Frame B: For terminase genes, 139 terminase accession numbers were used in the analysis. The type of environmental isolate and species are: C. botulinum (33), C. perfringens (2), C. tetani (14), soil (21), fecal-oral (43), waste water runoff (21), and thermophilic organisms (15). Low AA identity is seen for the majority of C. tetani TerL proteins. (TIF) [file pone.0182909.s002.tif]

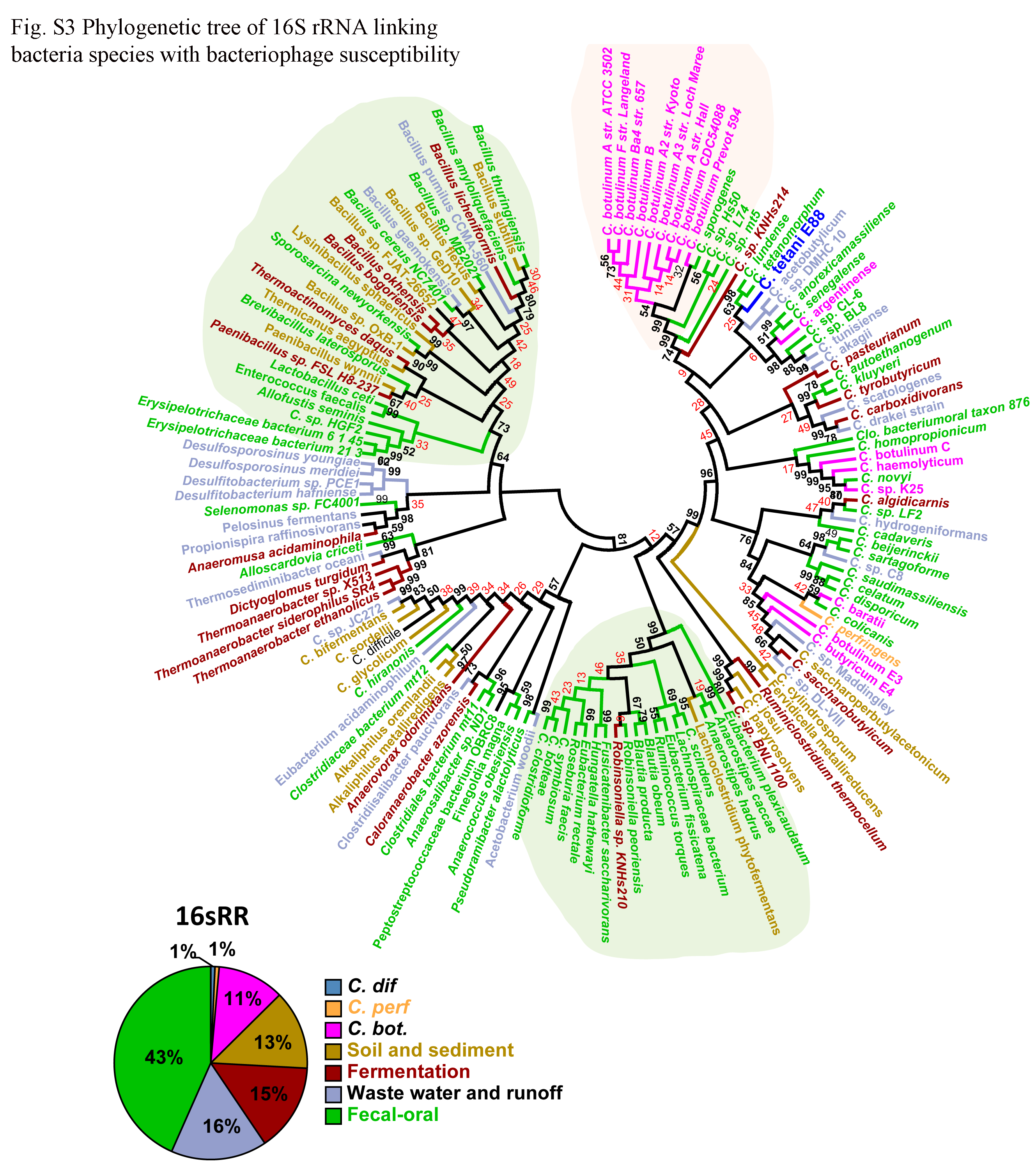

Supplement: S3 Fig — (A) Circular phylogenetic tree of 16sRR constructed from 144 bacterial species (417 phage and 22 CRISPR/Cas proteins). Multiple sequence alignment was performed using the MUSCLE algorithm with neighbor joining algorithm and phylogenetic trees were constructed by maximum likelihood analysis with bootstrapping (values <50 are in red). Bacterial species are color-coded based on type of environmental isolate or species: C. botulinum (16, magenta), C. perfringens (1, orange), C. tetani (1, blue), soil and sediment (19, light brown), fecal-oral (62, green), waste water and runoff (23, silver), and thermophilic organisms (21, red). The C. botulinum strains were clustered along the tree into Group I, Group II, and Group III families with the exception of several distantly related BoNT-expression organisms, C. argentinense (BoNT/G) and C. baratii (BoNT/F). C. tetani showed significant similarity to sequenced strains C. tetanomorphum and C. lundense (C. cochlearium JCM 1396, despite having higher 16sRR sequence identity, was not present as the genome has not been sequenced). (B) Distribution of 16sRR sequences based on environmental isolate (fecal-oral, waste-water and runoff, soil and sediment) or species (C. botulinum, C. difficile, C. perfringens). Of the 144 bacterial species identified with high overall conservation in phage and CRISPR proteins, 43% were bacterial organisms predominately found inhabiting the gastrointestinal tract and feces. Redundant 16sRR sequences and species names were removed to better show representation. See supplemental S3 Table for sequence and accession number. (TIF) [file pone.0182909.s003.tif]

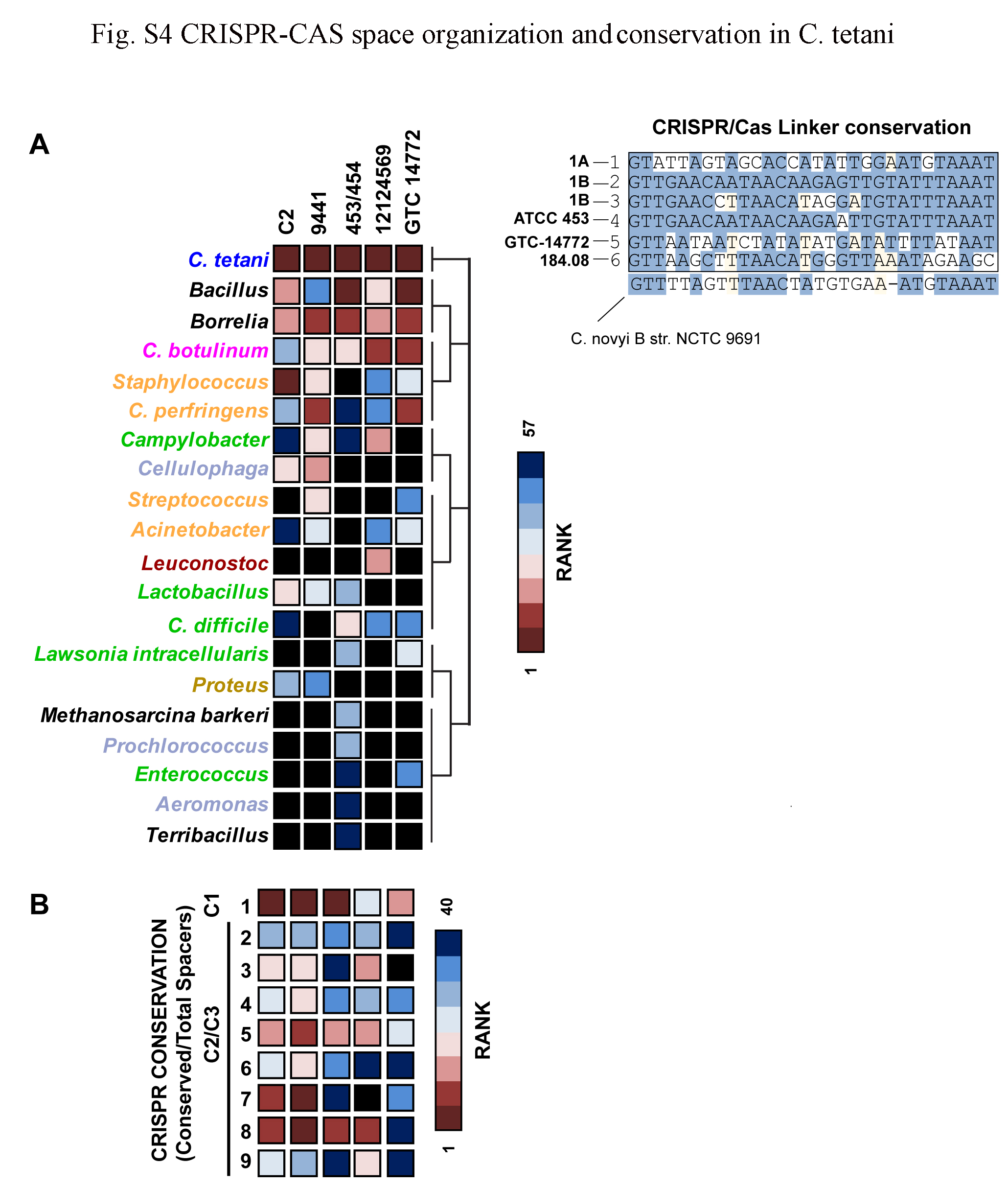

Supplement: S4 Fig — CRISPR/Cas arrays are conserved in C. tetani strains. CRISPR/Cas arrays were identified by conserved leader and linker sequences, and aligned against E88 reference genome. Conservation is shown as rank (1 = high conservation and 40 = low conservation) calculated by the number of conserved spacer sequences per total # spacers for each large array, I-A and I-B and Type III-A (in ATCC 453 and ATCC 454). CBER C2 is similar to ATCC 19406, CN655 and Strain A. Mean rank score were C2 (13.4), ATCC 9441 (13.2), ATCC 453 (26.60), 12124569 (20.9), and GTC 14772 (31.9). (TIF) [file pone.0182909.s004.tif]
