# Supplementary material for: Comparative pathogenomics of Clostridium tetani
Source: PLoS One. 2017 Aug 11;12(8):e0182909. doi: 10.1371/journal.pone.0182909 (PMC5553647; doi:10.1371/journal.pone.0182909)
Supplement: S3 Table — (DOCX) [file pone.0182909.s007.docx]

**Table 3 CRISPR Spacer Sequences**

| **Strain** | **CRISPR** | **CRISPR Linker sequence** | **#SP** | **Misc. features** |
| --- | --- | --- | --- | --- |
| **E88** | 9 | 1. GTATTAGTAGCACCATATTGGAATGTAAAT  2. GTTGAACAATAACAAGAGTTGTATTTAAAT  3. GTTGAACCTTAACATAGGATGTATTTAAAT | 34*  31  10* | C-1  C-2  C-2 |
| **C2** | 9 | 1. GTATTAGTAGCACCATATTGGAATGTAAAT  2. GTTGAACAATAACAAGAGTTGTATTTAAAT  3. GTTGAACCTTAACATAGGATGTATTTAAAT | 32  31  8 | C-1  C-2  C-2 |
| **19406** | 9 | 1. GTATTAGTAGCACCATATTGGAATGTAAAT  2. GTTGAACAATAACAAGAGTTGTATTTAAAT  3. GTTGAACCTTAACATAGGATGTATTTAAAT | 32  30  8 | C-1  C-2  C-2 |
| **CN655** | 9 | 1. GTATTAGTAGCACCATATTGGAATGTAAAT  2. GTTGAACAATAACAAGAGTTGTATTTAAAT  3. GTTGAACCTTAACATAGGATGTATTTAAAT | 32  30  7* | C-1  C-2  C-2 |
| **Strain A** | 9 | 1. GTATTAGTAGCACCATATTGGAATGTAAAT  2. GTTGAACAATAACAAGAGTTGTATTTAAAT  3. GTTGAACCTTAACATAGGATGTATTTAAAT | 32  31  8 | C-1  C-2  C-2 |
| **9441** | 9 | 1. GTATTAGTAGCACCATATTGGAATGTAAAT  2. GTTGAACAATAACAAGAGTTGTATTTAAAT  3. GTTGAACCTTAACATAGGATGTATTTAAAT | 34  30  5 | C-1  C-2  C-2 |
| **453** | 10 | 1. GTATTAGTAGCACCATATTGGAATGTAAAT  2. GTTGAACAATAACAAGAGTTGTATTTAAAT  3. GTTGAACCTTAACATAGGATGTATTTAAAT  4. GTTGAACAATAACAAGAATTGTATTTAAAT | 6  36  16  1 | C-1  C-2  C-2  C-3 |
| **454** | 10 | 1. GTATTAGTAGCACCATATTGGAATGTAAAT  2. GTTGAACAATAACAAGAGTTGTATTTAAAT  3. GTTGAACCTTAACATAGGATGTATTTAAAT  4. GTTGAACAATAACAAGAATTGTATTTAAAT | 6  36  17  1 | C-1  C-2  C-2  C-3 |
| **1214569** | 9 | 1. GTATTAGTAGCACCATATTGGAATGTAAATT  2. GTTGAACAATAACAAGAGTTGTATTTAAAT  3. GTTGAACCTTAACATAGGATGTATTTAAAT | 23  25  13 | C-1  C-2  C-2 |
| **GTC-14772** | 11 | 1. GTATTAGTAGCACCATATTGGAATGTAAAT  2. GTTGAACAATAACAAGAGTTGTATTTAAAT  3. GTTGAACCTTAACATAGGATGTATTTAAAT  5. GTTAATAATCTATATATGATATTTTATAAT | 11  44  10  7 | C-1  C-2  C-2  C-3 |
| **184.08** | 1 | **CRISPR System not present** | 3 | Absent |

Table S3. CRISPR/Cas arrays were analyzed by CRISPR finder (<http://crispr.u-psud.fr/>). Shown are repeat sequences for CRISPRs identified in *C. tetani* strains. The number of actual CRISPR arrays in each is likely lower because the majority of genome information is unfinished WGS data. For all strains with the exception of 184.08, ATCC 453/454, and GTC-14772, are two primary arrays with unique spacers (SP). A defective CRISPR/Cas system identified in strain 184.08, contained few spacer sequences.
