# Supplementary material for: Comparative pathogenomics of Clostridium tetani
Source: PLoS One. 2017 Aug 11;12(8):e0182909. doi: 10.1371/journal.pone.0182909 (PMC5553647; doi:10.1371/journal.pone.0182909)
Supplement: S5 Table — (DOC) [file pone.0182909.s009.doc]

**Table S5** PCR Primers

| **PCR target** | **Forward Primer** | **Reverse Primer** |
| --- | --- | --- |
| **TeNT** | GTGGAGCAACTACAAAATCAGC | AGCCAAAACCATCTCTACATGG |
| **RecA** | TCAACCAGATACAGGAGAACAAG | GCTTGAGACATCAATCTTGCTTG |
| **C2-Φ-A-IN** | AGAGGATTTAAAGCCATTTGAGG | ACGTGGAGATACCTTTGGAATAG |
| **9441-Φ-A-IN** | AGAGGATTTAAAGCCATTTGAGG | ACGTGGAGAAACCTTTGGAATAG |
| **453-Φ-A-IN** | AAGATACGATAGTGAAGATGAAGG | AGTATACTAAGGATAAACGTGGAG |
| **454-Φ-A-IN** | ATGCAGGTCAGATGGATGGAC | AGTATACTAAGGATAAACGTGGAG |
| **Universal Φ-B** | AGAATGTCTGTTATACCAGAAGATG | GAATGTTATGATAGGGCTTTAAGAG |
| **19406-Φ-C-IN** | TGAGGAACTAACTCAATCCAAGTC | ATAGACAAGCTTTCAAAGATAGTTTAG |
