# Supplementary material for: Comparative pathogenomics of Clostridium tetani
Source: PLoS One. 2017 Aug 11;12(8):e0182909. doi: 10.1371/journal.pone.0182909 (PMC5553647; doi:10.1371/journal.pone.0182909)
Supplement: S1 Appendix — (DOC) [file pone.0182909.s010.doc]

**S1 Appendix: Bacteriophage/Prophage Description**

*Prophage Genetic Conservation*

Notable differences between phages from insertion sites A, B and C were the DNA packaging enzyme (phage terminase, large subunit TERM), the type of site-specific DNA integrase or recombinase (tyrosine integrase, TYR or serine recombinase, SER), and portal protein (HK97 or SPP1 protein). Site-specific integration of lysogenic phages into host bacterial genomes occurs through DNA recombination mediated by a family of serine recombinases. 15 serine recombinase genes were identified in *C. tetani*.

Phages from strains C2 and ATCC 19406 were identical with the exception of Phage C from 19406. Corresponding integration sites in E88 reference strain are C2 Phage A: 1,138,018 - 1,185,037; C2 Phage B: 2,281,381 - 2,243,139; 19406 Phage C: 1,404,103 – 1,404,066. A leu-tRNA flanks C2 Phage B, 19406 Phage B, and 453 phage B insertion site at 2,243,062 - 2,243,147.

DNA packaging of the phage genome occurs through a conserved terminase complex, which consists of the recognition protein TerS, (terminase small subunit) and the endonuclease/translocase TerL (terminase large subunit) that processes viral DNA concatemers into DNA complexes that are fed through a portal protein vertex into empty phage proheads. Comparative analysis of phage portal and TerL proteins showed varying degrees of phage conservation based on genomic integration sites and serine recombinase homology (S2 and S3 Figs). Phylogenetic analysis of 147 portal proteins including 17 identified *C. tetani* portal proteins identified distinct clads based on SPP1, HK97, and nucleoid-structuring (H-NS) domain-containing proteins. High level of conservations (>95%) was found for SPP1-family portal proteins in *C. tetani* strains 12124569, 184.08, C2, and GTC-14772 in insertion site A and 12124569 and 184.08 in insertion site E. In contrast, portal proteins from the corresponding insertion site in ATCC 453 and ATCC941 possessed only 32% sequence identity and were significantly divergent from the other strains at this site (<20%). Less overall sequence conservation was observed for HK97-family portal protein in other *C. tetani* prophages, insertion site B (42% sequence identity between C2 and ATCC 453) and insertion site D (78% sequence identity between C2 and 184.08 and <29% for ATCC 453 and all other identified portal proteins). Two nucleoid-structuring proteins (H-NS portal proteins) identified within the same contig in strain 184.08 (WP_039262173.1 and WP_039261766.1) were distinct, sharing 84% identity with each other and low sequence identity to other portal proteins. Fecal-oral (27%) and fermenter (20%) bacterial strains were overrepresented in 147 sequences analyzed for sequence identity to *C. tetani* portal proteins.

TerL proteins showed a similar phylogenetic relationship observed for portal proteins (S3 Fig). C2 and 184.08 TerL proteins in insertion site A were identical and organized with ATCC 453 A and GTC-14772 A despite significantly lower conservation (<20%). Modest conservation was seen for TerL protein in ATCC 453-B (57%) and ATCC 453-D (52%) for ATCC 19406-C and C2-B, respectively. Phage TerL proteins showing moderate conservation to *C. tetani* TerL proteins were predominately fecal-oral bacterial strains (30%) and *C. botulinum* strains (24%). Sequences coding for TerL and TerS subunits were absent from strain 12124569 at insertion site A, suggesting that this prophage may be replication defective.

Tail tape measure protein (TTMP) is the major determinant of phage tail length; repeating units determine the length of the protein. Phylogenetic comparison of TTMPs from *C. tetani* strains showed moderate conservation by integration site. Shorter TTMPs (<700 aa) were present in strains C2, GTC-14772, ATCC 9441, and ATCC 453 compared to 184.08 and 12124569 (815 and 809 aa) in phage integration site A. Moreover, TTMPs from ATCC 9441 and ATCC 453 were 99% identical and shared high level of conservation with TTMPs identified in *C. botulinum* strains. In contrast, C2 and GTC-14772 (98% sequence identity) and 184.08 and 12124569 (100% sequence identity), did not show sequence similarity to other bacterial species or environmental isolates. Moderate sequence identity was found for TTMP at phage integration site B in strains C2, 184.08, and ATCC 453. TTMPs from C2 and ATCC 453 in IS-D were distinct (26% sequence identity). We identified three *C. perfringens* *Siphoviridae*-family phages phiSM101, phi3626, and phiS63 that shared homology to TTMPs in C2 IS-B, ATCC 453 IS-D, and ATCC 19406 IS-C. A large 2,070 aa phage protein was identified in GTC-14772 with modest sequence identity (<50%) to TTMPs in both plasmid and circular phages of group III *C. botulinum*, *C. novyi* and *C. haemolyticum*.

*Myoviridae*-like phage particles were observed by TEM for purified phage lysates from ATCC 453 (Fig. 7). The presence of contractile sheath proteins (e.g. XkdK) upstream of the PBSX XkdM-like major tail protein and TTMP are responsible for the long, contractile tails present in *Myoviridae* family phages. Homologous sequences to *Bacillus subtilis* prophage PBSX Xkd-family proteins that constitute the contractile tail were present in *C. tetani* phages at phage integration site B (phi-CT), despite the presence of contractile tail observed for only strain ATCC 453.

Phages isolated from strains C2 and ATCC 19406 appeared to be similar with electron-dense heads and long non-contractile tails with clear tail striations. Phages isolated from ATCC 19406 had longer and wider tails compared to C2, 18.5 ± 0.9 nm (n=18) versus 14.8 ± 0.7 nm (n=11) respectively (p < 0.01). Phage obtained from ATCC 9441 lacked tails, and possessed negatively stained phage heads. Phage obtained from ATCC 453 displayed distinct contractile, sheathed tails and hexagonal heads consistent with being in the viral family *Myoviridae*. Phage obtained from ATCC 453 had relatively short tails (125.3 ± 2.8 nm) compared to C2 (134.4 ± 4.1 nm) or ATCC 19406 (143.7 ± 3.5 nm) (significant by one-way ANOVA, F_2,40_ =7.944 p <0.001). Tail diameter was significantly greater for ATCC 453 (20.2 ± 0.7 , n=14) compared to phages obtained from strains C2 or 19406 (significant by one-way ANOVA, F_2,40_ =10.122, p <0.001).
