# Supplementary material for: Comparative pathogenomics of Clostridium tetani
Source: PLoS One. 2017 Aug 11;12(8):e0182909. doi: 10.1371/journal.pone.0182909 (PMC5553647; doi:10.1371/journal.pone.0182909)
Supplement: S2 Appendix — (DOC) [file pone.0182909.s011.doc]

**S2 Appendix: CRISPR-Cas Genetic Conservation**

The type I-A CRISPR complex (Fig. 1) was comprised of 8 predicted proteins (Cas6 – Cas8a1 - Cas7 - Cas5 - Cas3 - Cas4 - Cas1 - Cas2) and had significant homology and organization to proteins identified in diverse anaerobic gram-positive organisms [*Acetobacterium woodii DSM 1030* (62%, waste-water), *Anaerostipes caccae DSM 14662* (58%, gut), *C. akagii* DSM 12554 (63%, beach litter), *C. saratagoforme AAU1* (77%, bovine rumen), *Eubacterium sp. CAG:274* (59%, gut), and *Terrisporobacter glycolicus* (70%, soil sediment)]. Two predicted transposases were upstream of the type I-A CRISPR/Cas operon and a large CRIPSR array was downstream of Cas2. The type I-B complex (Fig. 1, F2) was comprised of 10 proteins (Cas2 - Cas1 - Cas4 - Cas3 - Cas5 - Cas7b – Cas8b1/Csh1 - Cas6 - Cas1 – Cas1) with significant homology to CRISPR/Cas proteins identified in many Clostridial species, *C. botulinum* (72%, BoNT/A type), *C. homopropionicum* (74%, sewage), *C. lundense* (68%, bovine rumen), *C. sp BL8* (66%, gut), *C. sp.* CL-6 (69%, gut), and *C. sp LF2* (67%, gut). In contrast to the type I-A CRISPR complex, CRISPR arrays were interspersed throughout the type I-B operon and showed very little homology [13].

A type III-A CRISPR/Cas system of 7 predicted proteins (Csm1 - Csm2 - Csm3 - Csm4 - Csm5 - Csx1 - Cas6) was found in China strains ATCC 453 and ATCC 454. Modest homology and organization was found for only 3 bacterial strains, *C. botulinum* CDC54088 (79% identity; Argentina: soil), *C. drakei* SL1 (47% identity; Germany, soil sediment), and *C. acetobutylicum* GXAS18-1 (46% identity; China, soil). Low sequence identity (22% - 48%, 34% average) was found for diverse thermophilic microrganisms (*Candidatus Acetothermus, Methanothermococcus thermolithotrophicus; Methanotorris igneus, and Thermosipho africanus*). An incomplete CRISPR/Cas system was identified in a GTC-14772 comprised of 5 predicted proteins (Cas3 – Cas5 – Cas7 – Cas8b/Csh1) with modest sequence identity (<40%) to a CRISPR/Cas Phage system in the plasmid for Group III, *C. bot* BKT015925.
